# Prevalence of SARS-CoV-2 infection among health care workers in a reference hospital in Brazil

**DOI:** 10.1590/S1678-9946202365052

**Published:** 2023-10-09

**Authors:** Vanessa Neves Almeida, Roberta Figueiredo Cavalin, Juliana Failde Gallo, Cleide Aparecida Guerra, Karen Cristina Rolim Madureira, Meire Bócoli Rossi, Rozania Soeli dos Santos Sobreira, Ana Paula Santos, Expedito Luna, José Angelo Lauletta Lindoso

**Affiliations:** 1 Instituto de Infectologia Emilio Ribas, São Paulo, São Paulo, Brazil; 2 Universidade de São Paulo, Faculdade de Medicina, Departamento de Medicina Preventiva, São Paulo, São Paulo, Brazil; 3 Universidade de São Paulo, Faculdade de Medicina, Departamento de Moléstias Infecciosas e Parasitárias, São Paulo, São Paulo, Brazil; 4 Universidade de São Paulo, Faculdade de Medicina, Instituto de Medicina Tropical de São Paulo, Laboratório de Protozoologia (LIM-49), São Paulo, São Paulo, Brazil

**Keywords:** SARS-CoV-2, Health care workers, Infection, Seroprevalence

## Abstract

Health care workers (HCW) are the frontline workforce for COVID-19 patient care and, consequently, are exposed to SARS-CoV-2 infection due to close contact to infected patients. Here, we evaluate the prevalence of SARS-CoV-2 infection among HCW from an infectious disease hospital, reference center for COVID-19 care in the metropolitan area of Sao Paulo city, Brazil. Among 2,204 HCW, 1,417 (64.29%) were subjected to detection of anti-SARS-CoV-2 antibodies by chemiluminescent immunoassay. Out of the total, 271 (19.12%) presented anti-SARS-CoV-2 antibodies. Prevalence varied according to HCW categories. The highest prevalence was observed in workers from outsourced companies, cooks and kitchen assistants, hospital cleaning workers, and maintenance workers. On the other hand, resident physicians and HCW from the institution itself presented lower prevalence (nurses, nursing assistants, physicians, laboratory technicians). Social and environmental factors are important determinants, associated with exposure in the hospital environment, which can determine the greater or lesser risk of infection by pathogens that spread rapidly by air.

## INTRODUCTION

COVID-19 is a new disease caused by SARS-CoV-2, transmitted human-to human^1-3^ mainly by respiratory route. However other transmission routes can occur, such as direct contact, or by contact with contaminated surfaces^3,4^. In the general population, the prevalence of SARS-CoV-2 infection ranged from 0.37% to 22.1% with a pooled estimate of 3.38%. In South America, the seroprevalence was estimated in 1.45%^2^. A seroprevalence survey in people aged over 18 years in the Sao Paulo city showed a 43.8% unadjusted prevalence^5^.

Health care workers (HCW), directly or indirectly exposed, are a vulnerable cohort to acquire infection transmitted by infected-patients^6,7^. HCW are the frontline workforce for COVID-19 patients care. Consequently, they are exposed to SARS-CoV-2 infection, due to close contact to infected-patients in different areas in the hospital^6^. The prevalence of SARS-CoV-2 infection in HCW varied according to region and methodology used to determine it. Some studies reported from 2.4% to 2.7% prevalence detected by PCR^8,9^. Using serological methods, the prevalence ranged from 11.2% to 24.4%^6,10,11^. Notably, the prevalence of asymptomatic HCW is a concerning aspect, since they can carry the virus and maintain the chain of transmission; some authors reported a prevalence of SARS-CoV-2 viral carriage around 2.4%^12^. However, among symptomatic HCW, the prevalence may reach 42.4%^7^. COVID-19 can present itself in different clinical forms, ranging from a flu-like syndrome to severe acute respiratory distress syndrome^3,13^. However, asymptomatic carriers, or detection of anti-SARS-CoV-2 antibodies, without previous symptoms has been reported^12,13^. Understanding the prevalence of SARS-CoV-2 antibodies and risk factors in HCW is important, to assess the effectiveness of individual protection measures^14^ that are being used in these health care institutions and identify possible immunized HCW.

In this study we evaluated the serological prevalence of SARS-CoV-2 infection in HCW from an infectious disease-specialized hospital, dedicated to treat COVID-19 patients in Sao Paulo’s metropolitan area, before vaccines were available.

## MATERIALS AND METHODS

### Study localization

The Institute of Infectology Infectologia Emilio Ribas (IIER), a reference hospital for infectious diseases in Sao Paulo city, Brazil, is composed by an outpatient and an inpatients ward. The main building encompasses the areas of imaging exams, endoscopies, the surgical center, the emergency department, two intensive care units and four wards. Approximately, 180 beds are distributed according to the level of care required by the patient. The intensive care unit has 40 beds and the wards 90 beds. The emergency room has 18 beds for emergency care. From March 2020 to mid 2021, the hospital was almost exclusively dedicated to COVID-19 patients care. In three other buildings, administrative health professionals worked without direct contact with patients.

### Study population and design

A cross-sectional study was performed at IIER, including HCW according to the following professional categories: administrative, cleaning workers, nursing, maintenance workers, physicians, and security workers. The following demographic and social data were obtained: sex, race/ethnicity, age (in years), municipality of residence, time spent commuting, type of transportation used to commute, schooling level, family income, loss of family income during pandemic and sector of work in the hospital. The hospital’s HCW were informed about the study. Those who were interested, attended the research center, where the study was presented to them. Those who agreed to participate were asked to sign an informed consent form, and blood collection was carried out. They were asked to access the study website to complete the questionnaire. Serum from HCW were collected from July 2020 to December 2020 before introduction of vaccination.

### Chemiluminescent immunoassay

Serum were subjected to detection of anti-SARS-CoV-2 antibodies, following the manufacturer’s recommendations. A commercial chemiluminescent immunoassay (Ortho Clinical Diagnostic, USA) was used to detect immunoglobulin G anti-spyke from SARS-CoV-2. The results are expressed in reactivity index (RI). A result was considered positive if RI ≥ 1. Quantitative Polymerase Chain Reaction (qPCR) was performed using an automated commercial platform (Cobas SARS-CoV-2 test, Roche diagnostics, Basel, Switzerland), based on two distinct N gene targets, as described by Centers for Disease Control and Prevention. The limit of detection is 250 copies/mL.

### Statistical analysis

Seroprevalence was determined by the proportion of positive results in the antibodies assay. Descriptive statistics were used to summarize the prevalence of detection of anti-SARS-CoV-2 antibodies according to HCW classification. The risk ratio and chi-squared test were calculated, considering one of the categories as reference. A p-value < 0.05 was considered significant. Statistical tests were performed using Epi Info™ version 7.1.4.0. Logistic regression analysis, via backward stepwise method, and Hosmer-Lemeshow’s test were performed using SPSS statistical package. Unadjusted and adjusted odds ratios were calculated for each variable in the model.

### Ethical statement

The study was approved by Research Ethical Committee from IIER (CAE 32264120.5.2001.0061).

## RESULTS

**Figure 1:**
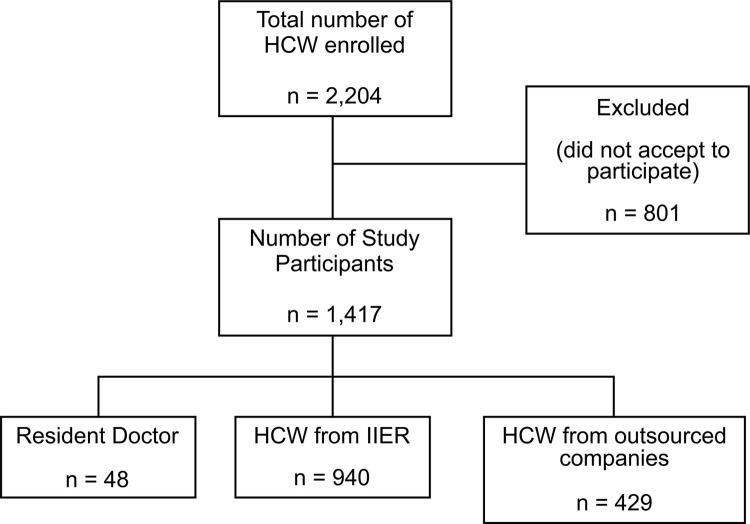
Flowchart of the schematic design of the seroprevalence study of SARS-CoV-2 infection in health workers at the Institute of Infectology Emilio Ribas during the COVID-19 pandemic, from July to December 2020.

From 2,204 eligible HCW, 1,417 (64.29%) were subjected to anti-SARS-CoV-2 antibodies detection, distributed in three categories: 48 resident physicians, 940 HCW from IIER and 429 workers from outsourced companies (Figure 1). A total of 271 (19.12%) HCW presented anti-SARS-CoV-2 antibodies (Table 1). Only 21 (9.58%) from the 271 individuals with IgG antibodies positive were symptomatic, and had the diagnostic of COVID-19 confirmed by qPCR, before the study. To better understand the distribution of seroprevalence of SARS-CoV-2 infection in this population, the participants were distributed according to categories. From 48 resident physicians, eight (16.7%) tested positive; 115 of 940 HCW from IIER were positive (12.2%), as were 148 of 429 (34.5%) professionals from outsourced companies (Table 1). HCW from outsourced companies presented a higher seroprevalence when compared to resident physicians and HCW from IIER.

**Table 1:** Seroprevalence of SARS-CoV-2 infection in health care workers at the Institute of Infectology Emilio Ribas, according to the employment relationship, July to December 2020.

Category	Number of HW included in the study	SARS-CoV-2 positive (n)	Seroprevalence % (95%CI)	p-value*
Resident physician	48	8	16.7 (8.1 – 29.2)	<0.0001
Health care workers from IIER*	940	115	12.2 (10.3 – 14.5)	
Outsourced companies workers**	429	148	34.2 (30.1 – 39.1)	
**Total**	**1,417**	**271**	**20.6 (18.7 – 22.7)**	

^*^physicians, nurses, nursing assistants, physical therapists, laboratory technicians; **physicians, nurses, nursing assistants, physical therapists, security workers, cooks and kitchen assistants, cleaning workers, maintenance workers, clerks, administrative workers.

According to professional categories, we observed a higher prevalence in technicians from outsourced companies, cooks and kitchen assistants, hospital cleaning workers, and maintenance workers. There were no differences between nurses, nursing assistants and technicians, physicians and laboratory technicians from IIER, resident physicians and security works (Table 2).

**Table 2:** Seroprevalence of SARS-CoV-2 infection in health care workers at the Institute of Infectology Emilio Ribas, according to professional category and activity, July to December 2020.

Profession and health work activity	Total number of HCW	Number of Participants	SARS-CoV-2 positive (n)	SARS-CoV-2 negative (n)	Seroprevalence (95%CI)	Risk ratio (95%CI)	p-value**
Frontline COVID-19 workers from IIER*	1,633	945	123	822	13.0 (10.9-15.2)	–	–
Resident physicians	88	49	9	40	18.37 (7.53-29.21)	1.41 (0.76-2.60)	0.3895
Security workers	61	61	8	53	13.11 (4.64-21.59)	1.01 (0.52-1.96)	0.8618
Frontline COVID-19 workers from outsourced companies**	237	193	72	121	37.31 (30.48-44.13)	2.87 (2.24-3.67)	<0.0011
Cooks and kitchen assistants	50	38	13	25	34.21 (19.13-49.29)	2.63 (1.64-4.21)	0.0005
Hospital cleaning workers	103	99	33	66	33.33 (24.05-42.62)	2.56 (1.85-3.54)	<0.0011
Building maintenance workers	32	32	13	19	40.62 (23.61-57.64)	3.12 (1.99-4.89)	<0.0011
Total	2,204	1,417	271	1146	19.12 (17.14-21.24)	1.58 (1.38-1.80)	<0.0001

^*^physicians, nurses, nursing assistants, physical therapists, laboratory technicians; **physicians, nurses, nursing assistants, physical therapists, clerks.

From 1,417 participants, 661 answered the form on demographic and social data. In the unadjusted analysis, Black or Mixed-race race/ethnicity, use of public transportation, complete secondary education or incomplete higher education and incomplete primary education, family income less than US$ 290.00 were the variables associated with the risk of SARS-CoV-2 infection (Table 3). In the adjusted model, Black or Mixed-race race/ethnicity (OR = 1.743; 95%CI 1.172-2.594), use of public transportation (OR = 2.134; 95%CI 1.247-3.652), to have complete secondary education or incomplete higher education (OR = 1.674; 95%CI 1.033-2.714), to have complete primary education or incomplete secondary education (OR = 1.298-5.462), and to be a hospital maintenance worker (OR = 3.356; 95%CI 1.248-9.026) were the variables independently associated to a positive serologic result (Table 4).

**Table 3:** Seroprevalence of SARS-CoV-2 infection in health care workers at the Institute of Infectology Emilio Ribas (n=661), according to demographic data, July to December 2020.

	Seroprevalence % (95%CI)		Serological Result				p-value*

			Positive (n=171)		Negative (n=490)		

			n	%	n	%	
**Sex**							0.758
Male	25.0	(18.8 – 32.1)	44	25.7	132	26.9	
Female	26.2	(22.3 – 30.3)	127	74.3	358	73.1	
**Ethnicity**							<0.001
White	19.6	(15.5 – 24.4)	64	37.4	262	53.5	
Black or Mixed-race	33.3	(28.1 – 38.9)	104	60.8	208	42.4	
Asian	13.0	(2.8 – 33.6)	3	1.8	20	4.1	
**Age**							0.658
60 years or more	22.1	(12.9 – 33.8)	15	8.8	53	10.8	
40 - 59 years	25.7	(21.5 – 30.2)	104	60.8	301	61.4	
18 - 39 years	27.7	(21.4 – 34.6)	52	30.4	136	27.8	
**Municipality of residence**							0.212
Sao Paulo	25.3	(21.5 – 29.3)	126	73.7	373	76.1	
Cities in the metropolitan area of Sao Paulo	28.6	(21.4 -36.6)	42	24.6	105	21.4	
Other	14.3	(1.8 – 42.8)	2	1.2	12	2.4	
No information	100.0	(2.5 – 100.0)	1	0.6		0.0	
**Time spent commuting**							0.108
Less than 30 minutes	18.5	(11.7 – 27.1)	20	11.7	88	18.0	
30 to 59 minutes	24.2	(18.7 – 30.4)	53	31.0	166	33.9	
60 to 119 minutes	28.5	(23.2 – 34.2)	78	45.6	196	40.0	
120 minutes or more	33.3	(21.7 – 46.7)	20	11.7	40	8.2	
**Transportation used to commute**							< 0.001
Individual transportation	14.0	(9.43 –19.7)	27	15.8	166	33.9	
Public transportation	30.8	(26.6 – 35.2)	144	84.2	324	66.1	
**Schooling**							< 0.001
Complete higher education or graduate studies	17.7	(13.8 – 22.3)	58	33.9	269	54.9	
Complete secondary education or incomplete higher education	30.6	(24.9 – 36.7)	77	45.0	175	35.7	
Complete primary education or incomplete secondary education	43.3	(30.6 – 56.8)	26	15.2	34	6.9	
Incomplete primary education	45.5	(24.4 – 67.8)	10	5.8	12	2.4	
**Family income**							< 0.001
> US$ 3,880.00	20.0	(7.7 – 38.6)	6	3.5	24	4.9	
US$ 1,940.00 - U$ 3,880.00	15.5	(7.5 – 27.4)	9	5.3	49	10.0	
US$ 1,164.00 - U$ 1,940.00	18.9	(10.8 – 29.7)	14	8.2	60	12.2	
US$ 873.00 - U$ 1,164.00	22.6	(14.6 – 32.4)	21	12.3	72	14.7	
US$ 582.00 - U$ 873.00	20.8	(14.2 – 28.8)	27	15.8	103	21.0	
US$ 290.00 – U$ 582.00	30.8	(24.2 – 38.0)	56	32.7	126	25.7	
< U$ 290.00	42.7	(32.6 – 53.6)	38	22.2	51	10.4	
No information	0.0	(0 – 52.2)	0	0.0	5	1.0	

^*^Chi-squared test.

**Table 4:** Results of the logistic regression analysis of factors associated to seroprevalence of SARS-CoV-2 IgG antibodies (n=661).

	Unadjusted OR	(95%CI)	p-value*	Adjusted OR	(95%CI)	p-value*
**Sex**						
Male	reference					
Female	1.064	(0.716-1.582)	0.758			
**Ethnicity**						
White	reference			reference		
Black or Mixed-race	2.047	(1.427-2.936)	<0.001	1.743	(1.172-2.594)	0.006
Asian	0.614	(0.177-2.130)	0.442	0.696	(0.195-2.486)	0.577
**Age**						
60 years or more	reference					
40 - 59 years	1.221	(0.660-2.258)	0.525			
18 - 39 years	1.351	(0.701-2.604)	0.369			
**Municipality of residence**						
Sao Paulo	reference					
Cities in the metropolitan area of Sao Paulo	1.184	(0.785-1.786)	0.420			
Other	0.493	(0.109-2.235)	0.359			
No information						
**Time spent commuting**						
Less than 30 minutes	reference					
30 to 59 minutes	1.405	(0.790-2.498)	0.247			
60 to 119 minutes	1.751	(1.008-3.041)	0.047			
120 minutes or more	2.200	(1.067-4.537)	0.033			
**Transportation used to commute**						
Individual transportation	reference			reference		
Public transportation	2.733	(1.740-4.292)	<0.001	2.134	(1.247-3.652)	0.006
**Schooling**						
Complete higher education or graduate studies	reference			reference		
Complete secondary education or incomplete higher education	2.041	(1.381-3.015)	<0.001	1.674	(1.033-2.714)	0.036
Complete primary education or incomplete secondary education	3.547	(1.978-6.361)	<0.001	2.662	(1.298-5.462)	0.008
Incomplete primary education	3.865	(1.594-9.373	0.003	2.719	(0.918-8.053)	0.071
**Family income**						
> US$ 3,880.00	reference					
US$ 1,940.00 - U$ 3,880.00	0.735	(0.234-2.303)	0.597			
US$ 1,164.00 - U$ 1,940.00	0.933	(0.321-2.713)	0.899			
US$ 873.00 - U$ 1,164.00	1.167	(0.421-3.229)	0.767			
US$ 582.00 - U$ 873.00	1.049	(0.390-2.822)	0.925			
US$ 290.00 – U$ 582.00	1.778	(0.689-4.589)	0.234			
< US$ 290.00	2.980	(1.109-8.007)	0.030			
No information						
**Loss of family income during pandemic**	1.146	(0.808-1.624)	0.445			
**Any family member lost their job during pandemic**	1.321	(0.919-1.899)	0.132			
**Obesity**	1.566	(1.069-2.293)	0.021			
**Cardiovascular disease**	1.349	(0.861-2.111)	0.191			
**Diabetes mellitus**	0.679	(0.321-1.437)	0.311			
**Pulmonary disease**	0.671	(0.249-1.809)	0.431			
**Workplace**						
Administrative	reference					
Healthcare	0.932	(0.657-1.323)	0.694			
**Professional category**						
Administrative	reference			reference		
Cleaning	2.665	(1.442-4.927)	0.002	1.392	(0.691-2.806)	0.355
Nursing	1.615	(0.962-2.712)	0.070	1.638	(0.958-2.801)	0.071
Maintenance	4.442	(1.704-11.578)	0.002	3.356	(1.248-9.026)	0.016
Physician	0.785	(0.384-1.606)	0.508	2.281	(0.975-5.336)	0.057
Other health professionals	1.112	(0.572-2.162)	0.754	1.871	(0.910-3.850)	0.089
Security	0.673	(0.215-2.109)	0.497	0.459	(0.142-1.477)	0.191

*Chi-squared test.

## DISCUSSION

In our study, which evaluated 1,417 HCWs from IIER, we observed a seroprevalence of 19.12% of anti-SARS-CoV-2 antibodies. The variables independently associated with the seropositivity were the black/mixed-ethnicity, the use of public transportation to commute to work, a lower educational level, and to be a hospital maintenance worker. The study population was composed by different categories, including not only HCWs directly in contact with infected-patients (physicians, resident physicians, nurses and nursing assistants), but we also included HCWs from other categories not directly exposed (kitchen assistants, security workers, cleaning workers, maintenance workers). Personal protective equipment (N95 mask, face shield, disposable apron, gloves, and protective goggles) were available to the HCW, irrespective of their professional categories. At the beginning of the SARS-CoV-2 pandemic, HCW were among the highest risk groups to acquire infection, due to their exposition to high viral load, taking care of patients infected with a new and unknown virus^2,14^. HCW exposed directly or indirectly to symptomatic or asymptomatic patients had an increased risk to be infected with SARS-CoV-2^15,16^. Data of seroprevalence of SARS-CoV-2 infection in HCW, including categories not directly involved in the patients’ care are scarce. Some reports have shown prevalence ranging from 2.4% to 38.9%, depending on population included and methods used in the diagnostic^9,16-18^. In general population, the prevalence of SARS-CoV-2 infection ranged from 5.1% to 5.7%. In a serological survey conducted in January 2021 in the Sao Paulo city, Brazil, a seroprevalence of 14.1% was identified among the adult population^5^. Lahner *et al*.^8^ observed a low prevalence of SARS-CoV-2 infection among HCWs, but higher than in the general population. Regarding figures among different categories, some points are remarkable, mainly when comparing COVID-19 front-line HCW from IIER with the workers from outsourced companies, working at the institution. Notably, seroprevalence of Sars-CoV-2 antibodies was almost three times lower in employees of the institution than in those from outsourced companies HCW (13.02% versus 37.31%). Some factors may have contributed to this significant difference. Firstly, after the onset of the pandemic, the IIER increased the number of ICU beds from 10 to 40, exclusively for COVID-19, and 30 of these beds were under the care of employees from outsourced companies. They could possibly be more exposed to COVID-19 infection than those in the institution, who would be treating fewer patients. However, HCWs from IIER worked in frontline at the wards and at the emergency unit, taking care of COVID-infected patients, which would place them in the same risk situation as HCWs from outsourced companies. According to demographic and social aspects the main variable associated to a higher prevalence of infection were Black or Mixed-race race/ethnicity, the use of public transportation, a lower educational level, and work in the hospital maintenance. Certainly, the longer exposure time on public transportation, a place of agglomeration, is one of the major factors that facilitate the spread of respiratory transmission pathogens, as in the case of SARs-CoV-2. Costa *et al.* ^16^ found 14% of seroprevalence in HCW from a tertiary hospital from Sao Paulo city and the main risk factors associated with infection were lower educational level, users of public transportation, and working in security and cleaning. In another study conducted in Latin America, lower socioeconomic strata were also associated with seropositivity among HCW^17^. Differently, security workers presented lower seroprevalence when compared to cleaning and nurses or nursing assistant from outsourced company in our study. In our cohort, risk of infection may be more related to outside environments other than the hospital. Since the first cases in the institution, a continuing education program was immediately instituted, for the use of personal protective equipment, as well as offering such equipment to all professionals, including outsourced company workers. Certainly, working continuously in an exclusive care unit for infectious diseases favors adherence to individual protection measures. The prevalence of COVID-19 according to human development index (HDI) was investigated in Sao Paulo city, where the hospital is located, and the lower HDI areas presented a 22.0% prevalence, while in the higher HDI areas it was 11.9%, corroborating the social determination of COVID-19 prevalence and morbimortality^5,20^. Oliveira *et al*.^19^ observed a 5.5% prevalence of SARS-CoV-2 infection, and the risk factor associated to infection was cleaning workers, with no relation with working directly in COVID-19 care units. To better understand the real prevalence in HCWs, we strongly recommend future studies to include different categories, those directly and indirectly exposed to SARs-CoV-2 infection. Social and environmental factors are important determinants, associated with exposure in the hospital environment, which can determine the greater or lesser risk of infection by pathogens that spread rapidly by air. Knowing the socioeconomic aspects and the habits of workers may help to conduct policies aimed at reducing the risk of infection in such a vulnerable population.

As limitations of our study, we point out that the study was carried out in a self-selected sample of HCW, although a high proportion of the eligible subjects participated in the serologic screening. Despite of the small proportion of online respondents, we considered the sample representative, because the distribution of the online respondents, regarding age and sex, is similar to the participants of the serosurvey.

## CONCLUSION

In conclusion, we observed a relatively high seroprevalence of anti-SARS-CoV-2 antibodies in our sample of HCW working in an infectious diseases reference hospital. Our analysis suggests that exposure outside the working environment was a more important risk factor of SARS-CoV-2 infection than the occupational exposure.
